# SEOM clinical guideline for treatment of muscle-invasive and metastatic urothelial bladder cancer (2018)

**DOI:** 10.1007/s12094-018-02001-x

**Published:** 2018-12-18

**Authors:** A. González del Alba, G. De Velasco, N. Lainez, P. Maroto, R. Morales-Barrera, J. Muñoz-Langa, B. Pérez-Valderrama, L. Basterretxea, C. Caballero, S. Vazquez

**Affiliations:** 1 Medical Oncology Department, Hospital Universitario Puerta de Hierro-Majadahonda, Joaquin Rodrigo 2, 28222 Majadahonda, Madrid Spain; 2 Medical Oncology Department, Hospital Universitario Doce de Octubre, Madrid, Spain; 3 Medical Oncology Department, Complejo Hospitalario de Navarra, Pamplona, Spain; 4 Medical Oncology Department, Hospital de la Santa Creu i Sant Pau, Barcelona, Spain; 5 Medical Oncology Department, Vall d’Hebron Institute of Oncology, Vall d’ Hebron University Hospital, Barcelona, Spain; 6 Medical Oncology Department, Hospital Universitari I Politècnic la Fe, Valencia, Spain; 7 Medical Oncology Department, Hospital Universitario Virgen del Rocio, Sevilla, Spain; 8 Medical Oncology Department, Hospital Donostia-Donostia Ospitalea, Donostia, Spain; 9 Medical Oncology Department, Ciberonc, Centro de Investigación Biomédica en Red Cáncer. Hospital General Universitario de Valencia, Valencia, Spain; 10 Medical Oncology Department, Hospital Universitario Lucus Augusti, Lugo, Spain

**Keywords:** Bladder cancer, Cystectomy, Chemotherapy, Immune checkpoint inhibitors

## Abstract

The goal of this article is to provide recommendations about the management of muscle-invasive (MIBC) and metastatic bladder cancer. New molecular subtypes of MIBC are associated with specific clinical–pathological characteristics. Radical cystectomy and lymph node dissection are the gold standard for treatment and neoadjuvant chemotherapy with a cisplatin-based combination should be recommended in fit patients. The role of adjuvant chemotherapy in MIBC remains controversial; its use must be considered in patients with high-risk who are able to tolerate a cisplatin-based regimen, and have not received neoadjuvant chemotherapy. Bladder-preserving approaches are reasonable alternatives to cystectomy in selected patients for whom cystectomy is not contemplated either for clinical or personal reasons. Cisplatin-based combination chemotherapy is the standard first-line protocol for metastatic disease. In the case of unfit patients, carboplatin–gemcitabine should be considered the preferred first-line chemotherapy treatment option, while pembrolizumab and atezolizumab can be contemplated for individuals with high PD-L1 expression. In cases of progression after platinum-based therapy, PD-1/PD-L1 inhibitors are standard alternatives. Vinflunine is another option when anti-PD-1/PD-L1 therapy is not possible. There are no data from randomized clinical trials regarding moving on to immuno-oncology agents.

## Introduction

According to GLOBOCAN, 2018 will witness some 549,000 new bladder cancer diagnoses and 200,000 bladder cancer deaths worldwide, making it the 10th most common type of cancer for both genders [1]. It is approximately four times more common in males as opposed to females, with incidence and mortality rates of 9.6 and 3.2 per 100,000, respectively, in men.

Europe has one of the highest incidence rates of bladder cancer in the world. According to cancer registry data, incidence rates in both sexes are highest in Southern Europe (Greece, having the highest incidence rate; Italy and Spain, with a total of 21,093 estimated new cases in 2015 [2]; Western Europe (The Netherlands and Belgium) and Northern America.

Cigarette smoking is the main risk factor for urothelial bladder with 50% of bladder cancer cases attributable to smoking in both sexes (ever-smokers have a 2.5 times higher risk of developing this tumor versus never-smokers). The second most important risk factor is occupational exposure to aromatic amines, polycyclic aromatic hydrocarbons, and chlorinated hydrocarbons [3].

## Methodology

The SEOM guidelines have been elaborated with the consensus of ten genitourinary cancer oncologists from Spanish Society of Medical Oncology (SEOM) and Spanish Oncology Genitourinary Group (SOGUG). To assign a level of evidence and grades of recommendation, we have used Table 1. Statements without grading were deemed justified standard clinical practice by the SEOM/SOGUG faculty and experts.

**Table 1:** Levels of evidence/grades of recommendation

Levels of evidence
(I) Evidence from at least one large randomized, controlled trial of good methodological quality (low potential for bias) or meta-analyses of well-conducted randomized trials without heterogeneity
(II) Small randomized trials or large randomized trials with a suspicion of bias (lower methodological quality) or meta-analyses of such trials or of trials with demonstrated heterogeneity
(III) Prospective cohort studies
(IV) Retrospective cohort studies or case–control studies
(V) Studies without control group, case reports, expert opinions
Grades of recommendation
(A) Strong evidence of efficacy with a substantial clinical benefit; strongly recommended
(B) Strong or moderate evidence of efficacy but having limited clinical benefit; generally recommended
(C) Insufficient evidence of efficacy or benefit; does not outweigh risk or disadvantages; optional
(D) Moderate evidence against efficacy or of adverse outcome; generally not recommended
(E) Strong evidence against efficacy or of adverse outcome; never recommended

## Molecular biology and classification

Urothelial bladder cancer is a heterogeneous epithelial malignancy with variable clinical outcomes. From a normal urothelium, loss of heterozygosity (LOH) of chromosome 9 has been associated with most urothelial cancers [4] with two main pathways for their development:

- *Non-muscle-invasive urothelial carcinomas* characterized by activation of the receptor tyrosine kinase-Ras pathway, activating mutations in HRAS or fibroblast growth factor receptor 3 (FGFR3) genes. FGFR3 and HRAS mutations are not generally present within the same cancer.
- *Muscle-invasive urothelial carcinoma* characterized by alterations in the p53 and retinoblastoma (RB1) pathways. These genes interact with the Ras-mitogen-activated protein kinase (MAPK) signal transduction pathways.

The most comprehensive molecular analysis of muscle-invasive bladder cancer has been provided by The Cancer Genome Atlas Project (TCGA), which has recently updated the study with 412 cases [5]. Tumors were categorized histologically and evaluated via whole genome sequencing, whole exome sequencing, DNA copy number, complete mRNA and microRNA expression, DNA methylation, protein expression, and phosphorylation.

- Fifty-eight genes were significantly mutated; these genes included TP53, KTDM2D, KDM6A, PIK3CA, RB1, and FGFR3. Mutations in the p53/RB tumor suppressor pathway were seen in nearly 90% of tumors and alterations in the PI3K/AKT/mTOR and RTK/RAS signaling pathways were observed in 71%. MIBC exhibits high overall mutation rates, which appears to be associated with mutation signatures for an endogenous mutagenic enzyme, APOBEC cytidine deaminase [6]. Neoantigen load displays a correlation with mutation burden and has been linked survival [7, 8].
- FGFR3-TACC3 was the most common gene fusion reported [9].
- Epigenetic changes were observed in nearly 90% of tumors.

### mRNA expression-based molecular subtypes

Several studies have proposed a molecular classification of bladder cancer based on the whole genome mRNA expression profile. The molecular subtypes identified in bladder cancer reveal significant similarities with the molecular classification previously established in breast cancer [10].

- The University of North Carolina group reported a classification of high-grade, muscle-invasive bladder tumors, in which they detected two main subtypes:(KRT5/6 and CD44) and luminal (PPARG, GATA3, KRT20, and UPK2). A 47-gene signature (BASE 47) classified high-grade bladder cancer in luminal and basal-like tumors [11].
- The MD Anderson group identified three distinct clusters: basal, luminal, and p53-like tumors [12].
- The Lund group identified two major molecular subtypes, designated MS1 and MS2, displaying differences in the number of genomic alterations, including F*GFR3* and *TP53* mutations [13].
- The TCGA study identified five expression subtypes [5]:
  - *Luminal-papillary* enriched with FGFR3 alterations; papillary histology.
  - *Luminal-infiltrated* characterized by the presence of lymphocytic infiltrates and chemoresistance. These tumors had increased expression of several immune markers, including PD1/PDL1. The wild-type p53 is also present in this subgroup.
  - *Luminal* highest expression levels of several uroplakins.
  - *Basal-squamous* basal and stem-like markers and squamous differentiation markers. More common in females.
  - *Neuronal* high expression of neuronal differentiation and development genes.

In conclusion, the TCGA study confirmed the existence of luminal (KRT20+, GATA3+, FOXA1+) and basal (KRT5,6,14+, GATA3−, FOXA1−) transcriptional subtypes, as well as identifying luminal and neuronal subtypes. The subtypes were associated with overall survival. Intrinsic subtypes in MIBC patients are associated with specific clinical–pathological characteristics.

## Clinical prognostic factors

Prognostic factors generally reflect tumor biology and the extent of disease and can be used to guide treatment decisions. For patients with non-muscle invasive bladder cancer, tumor histological grade is the single most important prognostic factor [14]. In MIBC, prognosis is derived from staging; i.e., whether the tumor is organ-confined (≤ T2) or non-organ-confined (≥ T3 and N +). Pathological stage establishes different prognostic categories for those undergoing radical cystectomy. The 5-year overall survival (OS) for ≤ T1 and pT1 tumors was 85% and 76%, respectively, while individuals with MIBC pT2, pT3a/pTb, and pT4 lymph node-negative tumors had 5-year OS rates of 77%, 64/49%, and 44%, respectively. Thus, 5-year OS of subjects with lymph node-negative tumors was significantly higher than those with positive lymph nodes (69% vs 31%, *P *< 0.001) [15]. Furthermore, complete pathological response following neoadjuvant chemotherapy is associated with improved OS [16].

The presence of visceral metastases (i.e., pulmonary, liver, bone) and poor performance status (Karnofsky Performance Status of ≤ 80%) were independent prognostic factors of poor OS following treatment with MVAC (methotrexate, vinblastine, doxorubicin, and cisplatin) in the first-line setting for advanced/metastatic disease. Median OS for patients who had zero, one, or two negative prognostic factors were 33, 13.4, and 9.3 months, respectively [17]. These factors have also been validated for newer combination chemotherapy regimens [18, 19].

Similar to the first-line setting, the presence of liver metastases, hemoglobin levels < 10 g/dl, and ECOG PS > 0 appear to predict worse outcomes in the second-line therapy for advanced/metastatic bladder cancer. Four subgroups were identified based on the presence of zero, one, two, or three adverse prognostic factors; the median OS times for these groups were 14.2, 7.3, 3.8, and 1.7 months (*p* < 0.001), respectively [20]. Additionally, shorter interval from prior chemotherapy appears to be an independent unfavorable prognostic factor [21].

Emerging data exploring the programmed cell death-1 protein (PD-1)/PD-1 ligand (PDL-1) checkpoint inhibitors have shown that ECOG PS 0 and 0 visceral metastases could be predictive for response to immune checkpoint inhibitors [22].

### Recommendations

Use of prognostic classification in first-line chemotherapy: Level of evidence II. Grade of recommendation B.

Use of prognostic classification in second-line chemotherapy: Level of evidence II. Grade of recommendation: B.

## Neoadjuvant and adjuvant treatment

The gold standard for patients with MIBC is radical cystectomy (RC) with extended lymphadenectomy and orthotopic urinary diversion [23, 24]. Nevertheless, some 50% of patients with MIBC will develop metastatic disease after undergoing RC, and only 25–35% with pT3–pT4 tumors (and/or with malignant lymph node involvement) are still alive 5 years after surgery. The goal of perioperative chemotherapy is to treat micrometastatic disease and avoid relapse.

### Neoadjuvant treatment

Several randomized trials have explored the benefit of neoadjuvant chemotherapy, although some of them have failed to show a clear benefit of this strategy [25]. Two large, randomized trials have exhibited survival benefit with cisplatin-based combination chemotherapy compared to surgery alone in patients with clinical stage cT2-T4aN0M0 who are candidates for RC or definite radiotherapy [16, 26]; moreover, two meta-analyses have confirmed this advantage, with a 13% reduction of risk of demise and a 5% absolute survival benefit at 5 years [27, 28]. There is no evidence that favors a single superior cisplatin-based neoadjuvant regimen. The two largest randomized trials used CMV (cisplatin, methotrexate, and vinblastine) or MVAC (cisplatin, methotrexate, adriamycin, and vinblastine) [16, 26]. Two prospective phase II trials have evaluated dose-dense (ddMVAC) in the neoadjuvant setting with encouraging results [29, 30]. In retrospective studies, CG (cisplatin/gemcitabine) has demonstrated complete pathological response (pCR) rates similar to those of MVAC, albeit with a better toxicity profile [31], and ddMVAC provides higher (pCR) and improved survival rates with respect to CG [32]. A comparative effectiveness study conducted by Galsky et al., found no significant differences in pCR rates between CG, MVAC, and ddMVAC [33]. There is insufficient data with any other cisplatin chemotherapy regimens for unfit patients in the neoadjuvant setting.

#### Recommendations

Neoadjuvant chemotherapy with cisplatin-based combination chemotherapy is recommended for T2-T4aN0M0 bladder cancer: Level of evidence I. Grade of recommendation A.

Data concerning individuals who are unfit for cisplatin are insufficient to provide a recommendation; therefore, in these patients, neoadjuvant chemotherapy is not recommended: Level of evidence I. Grade of recommendation A.

### Adjuvant treatment

The role of adjuvant chemotherapy in MIBC remains controversial. Few randomized studies have addressed this issue and the vast majority of trials that have included a small number of patients, were prematurely closed due to slow accrual or had methodological flaws. Four recent randomized trials have compared adjuvant chemotherapy with observation after RC in cases of MIBC. The first study looked at 114 patients with p53-altered pT1-T2N0 MIBC treated with MVAC. Neither the prognostic value of p53 nor the benefit of MVAC chemotherapy in patients with p53-positive tumors was confirmed [34]. An Italian study included 194 pT2-grade 3 or pT3–T4 patients and examined the effect of CG, but was underpowered to demonstrate a benefit in OS or progression-free survival (PFS) [35]. The third trial, conducted by SOGUG, appraised 142 subjects treated with CGP (cisplatin/gemcitabine/paclitaxel) and displayed a difference in 5-year survival favoring the chemotherapy arm (60% vs 31%; *p *< 0.001) [36]. Finally, the European Organization for Research and Treatment of Cancer (EORTC) 30994 study included 284 high-risk patients, who were randomized to receive adjuvant chemotherapy either immediately after RC or deferred until relapse. PFS was longer with immediate versus deferred adjuvant chemotherapy [Hazard ratio (HR): 0.54; *p *< 0.001], but no differences in OS were observed (HR 0.78; *p *= 0.13) [37].

A recent systematic review, including a meta-analysis of nine randomized trials with a total of 945 patients, demonstrated a benefit in OS for adjuvant chemotherapy over placebo (HR 0.77; *p *= 0.05). A benefit was also detected for relapse-free survival (HR 0.66; *p *= 0.01) that was even more evident in patients with lymph node involvement [38]. A combined analysis of this meta-analysis with the results of the EORTC study confirms this benefit in OS (HR 0.77; *p* = 0.002) favoring adjuvant chemotherapy [37]. There is as yet no data with respect to other chemotherapy regimens for individuals who are unfit for cisplatin.

#### Recommendations

For patients with high-risk MIBC (pT3–pT4 and/or lymph node involvement), who are able to tolerate a cisplatin-based regimen, and have not received neoadjuvant chemotherapy, adjuvant therapy with cisplatin-based chemotherapy after radical cystectomy is recommended: Level of evidence I. Grade of recommendation A.

For patients unfit for cisplatin, adjuvant chemotherapy is not recommended: Level of evidence I. Grade of recommendation A.

## Bladder-sparing treatments

Bladder-preserving approaches are reasonable alternatives to cystectomy for patients who are medically unfit for surgery and those who wish to avoid radical surgery.

Although there are no absolute criteria, key factors for identifying candidates for bladder preservation include urothelial histology, unifocal tumors < 5 cm, the absence of carcinoma in situ, maximal transurethral resection of bladder tumor (TURBT, early tumor stage (T2–T3a), no hydronephrosis, and good bladder function and capacity. Advanced age is not a contraindication for a multi-modality approach [39].

An appropriate alternative to cystectomy is TURBT followed by radiation therapy with concurrent chemotherapy. No definitive randomized trials have been completed that compare bladder-preserving trimodality treatment (TMT) with radical cystectomy [40]. A meta-analysis based upon data from 9000 patients in eight studies found no significant difference in overall survival, disease-specific survival, or progression-free survival at 5 or 10 years [41]. Other approaches include TURBT alone, TURBT followed by chemotherapy, TURBT followed by radiotherapy, and partial cystectomy. However, none have an established role in muscle-invasive bladder cancer.

TMT includes maximal TURBT followed by concurrent chemoradiotherapy, radiation (40–45 Gy to the pelvis) with concurrent radiosensitizing chemotherapy and an additional radiation boost to the bladder (20–25 Gy), if complete response is documented on repeat biopsy. If residual disease is present at response evaluation, surgical consolidation (salvage cystectomy) is recommended.

The benefit of adding chemotherapy to RT compared to RT alone is supported by two randomized trials. In the first randomized, phase III trial conducted in 360 patients, radiotherapy with concurrent mitomycin C and 5-FU improved 2-year locoregional disease-free survival (DFS) from 54% (radiotherapy alone) to 67% (HR 0.68), and 5-year OS from 35 to 48% (HR 0.82), without increasing grade 3–4 acute or late toxicity [42]. The second study of 99 patients randomized to receive radiation with or without cisplatin demonstrated a statistically significant decrease in the incidence of first recurrence in the pelvis with the addition of cisplatin [43]. The optimal chemotherapy regimen had not been defined in adequately powered randomized clinical trials.

Neoadjuvant chemotherapy in TMT has not been shown to improve survival. A phase III trial compared the efficacy of two cycles of CMV followed by concurrent chemoradiotherapy *vs* concurrent chemoradiotherapy alone. No difference in complete clinical response or 5-year OS was observed [44].

RTOG pooled analysis has demonstrated the effectiveness of this approach. This analysis included 468 patients who had clinical T2 to T4a tumors and a median follow-up of 4.3 years. The main results included [45]:

- 5-year and 10-year OS rates of 57 and 36%, respectively.
- Complete response rate following chemoradiotherapy was 69%.
- Of the 205 patients alive at 5 years, 80% had an intact bladder.

### Recommendations

TURBT alone or radiotherapy alone cannot be recommended as standard treatment: Level of evidence: II. Grade of recommendation: B.

TMT is an alternative in well-informed and compliant patients for whom cystectomy is not considered for clinical or personal reasons: Level of evidence: I. Grade of recommendation: A.

## Treatment of locally advanced or metastatic bladder cancer

### First-line therapy for fit patients

Cisplatin-based combination chemotherapy represents the standard of care for patients with metastatic disease. Combination therapy is more effective than single-agent cisplatin therapy alone. In a prospective randomized trial, MVAC was superior to single-agent cisplatin with respect to RR, duration of remission, and OS (12.5 vs. 8.2 months; *p *= 0.002) [46].

The EORTC conducted another randomized trial [47] that assessed the efficacy of a high-dose intensity MVAC regimen with a classic MVAC regimen. There were 21% CRs on the HD-MVAC arm and 9% on the MVAC arm (*p *= 0.009); the ORR was 62% and 50%, respectively (*p *= 0.06). PFS was significantly better with HD-MVAC (9.1 vs 8.2 months). An update at a median follow-up of 7.3 years reported that the HD-MVAC regimen was associated with improved OS (HR, 0.76; 95% CI 0.58–0.99; *p *= 0.042). It seems reasonable to reserve HD-MVAC for good prognosis and fit patients and when a rapid tumor response is needed.

Another combination therapy often used to treat advanced urothelial cancer (UC) in the first-line setting is gemcitabine and cisplatin (GC), which was evaluated in a multicenter, randomized, phase III trial that compared GC with the MVAC regimen in 405 patients with advanced or metastatic bladder cancer. GC yielded response rates, time-to-progression, and OS (HR 1.09; 95% CI 0.88–1.34; *p *= 0.66) that were similar to MVAC [48], although GC had a better safety profile and was better tolerated than MVAC. It therefore tends to be the preferred choice for first-line therapy.

In a randomized phase III trial, the combination of paclitaxel, cisplatin, and gemcitabine (PCG) was compared with GC. PCG did not show statistically significant differences in PFS or OS, and was associated with higher toxicity [49].

#### Recommendations

For first-line fit patients, both CG and MVAC are considered standard options. CG is preferred over MVAC, mainly due to its better safety profile: Level of evidence 1. Grade of recommendation A.

Early palliative care is strongly recommended.

### First-line therapy for unfit patients

A significant percentage of patients with advanced UC are considered ‘‘unfit’’ for cisplatin-based chemotherapy. Given the great variability in the definition of “unfitness for cisplatin”, it has been suggested that the definition adopted in clinical trials should be: ECOG PS 2, or Karnofsky PS of 60–70%, creatinine clearance < 60 mL/min, audiometric hearing loss, and/or peripheral neuropathy ≥ grade 2, CTCAE version 4.0, or NYHA class III heart failure [50].

An EORTC randomized phase II/III trial comparing the combination of carboplatin and gemcitabine (GCa) with methotrexate, carboplatin, and vinblastine (M-CAVI) in 238 unfit patients, found similar efficacy for the two regimens with a median OS of 9.3 and 8.1 months, respectively (*p *= 0.64), and lower toxicity for GCa (9.3% vs 21%) [19].

KEYNOTE-052 [51] is a single-arm, phase II, multicenter trial of pembrolizumab in 370 treatment-naïve, cisplatin-ineligible, subjects with locally advanced or metastatic UC. The ORR was 29%. Median OS was 11.5 months (95% CI 10.0–13.3) Overall, 67.6% of patients reported AEs of any grade. Most common AEs were fatigue (18.1%) and pruritus (17.8%). Immune-mediated AEs occurred in 24.6% of the sample.

The IMvigor 210 is a phase II, single-arm, two-cohort, multicenter trial of atezolizumab in locally advanced and metastatic UC: cohort 1 comprised patients ineligible for cisplatin-based chemotherapy as first-line treatment. After a median follow-up of 17.2 months, ORR was 23% for the entire cohort (28% for IC 2/3 group). Responses were durable with median duration of response (DOR) was not reached. Median PFS was 2.7 months, while median OS was 15.9 months. AEs were reported in 66% of patients; 16% exhibited grade 3–4 AEs. The most common AEs were fatigue (30%), diarrhea (12%), and pruritus (11%) [52].

Preliminary data from two phase III clinical trials (Keynote-361 and IMvigor130) show reduced survival with pembrolizumab and atezolizumab when used as first-line treatments for UC in patients with low levels of PD-L1. Atezolizumab and pembrolizumab should only be used for first-line treatment of UC in patients with high PD-L1 levels [53, 54].

#### Recommendations

For first-line treatment in unfit patients, GCa should be considered the preferred chemotherapy treatment option: Level of evidence 1. Grade of recommendation A.

Pembrolizumab and atezolizumab could be considered in patients with high PD-L1 expression levels: Level of evidence 3. Grade of recommendation B.

Early palliative care is strongly recommended.

### Second-line therapy

Until relatively recently, we have had limited treatment options for this scenario. Most of chemotherapy agents have been tested in phase II studies. Paclitaxel, docetaxel, oxaliplatin, pemetrexed, nab-paclitaxel, ifosfamida, among others, have a response rate of around 20%, without providing benefit in overall survival [55]. The use of a combination of chemotherapy agents increases response rates and DFS, albeit but not OS [56]. Vinflunine, a third-generation vinca alkaloid, demonstrated an OS benefit in eligible subjects during a phase III study, compared to the best supportive care (BSC). The results showed modest activity (overall response rate was 8.6%), a clinical benefit with a favorable safety profile, and survival benefit in favor of vinflunine with median OS of 6.9 vs 4.3 month. VFL reduced the risk of death by 22% compared to BSC (HR 0.78) (although not in the intended treatment population) and it has been approved by the European Medicines Agency (EMA) for this indication [57].

In the last 2 years, the EMA has approved the PD-L1 inhibitor atezolizumab, as well as the PD-1 inhibitors nivolumab and pembrolizumab for the treatment of locally advanced or metastatic urothelial cell carcinoma that has progressed during or after platinum-based chemotherapy regardless of PD-L1 expression levels.

An open-label, randomized, phase III trial compared pembrolizumab versus standard chemotherapy (paclitaxel, docetaxel, or vinflunine) in 542 patients with advanced urothelial carcinoma that recurred or progressed after platinum-based chemotherapy and revealed longer median OS for pembrolizumab-treated patients compared to chemotherapy (10.3 vs. 7.4 months; *p *= 0.002). Furthermore, fewer grade 3, 4, or 5 treatment-related adverse events (AEs) ensued in the group that received pembrolizumab with respect to those treated with chemotherapy (15.0% vs 49.4%) [58].

A phase II trial in locally advanced or metastatic urothelial carcinoma that progressed after at least 1 platinum-containing regimen reported an overall objective response in 52 of 265 patients (19.6%; 95% CI 15.0–24.9) after treatment with nivolumab. Median OS was 8.74 months (95% CI 6.05–not yet reached). Based on PD-L1 expression of < 1% and ≥ 1%, OS was 5.95 and 11.3 months, respectively [59].

Data from a 2-cohort, multicenter, phase II trial that evaluated atezolizumab in 310 patients with metastatic urothelial carcinoma post-platinum treatment yielded a significantly improved overall response rate compared to historical controls (15% vs. 10%; *p *= 0.0058); at median follow-up of 11.7 months, these responses have shown to be durable with good tolerability [60]. The phase III IMvigor211 study that appraised atezolizumab compared with standard chemotherapy failed to meet its primary endpoint of OS in cases of high expression of PD-L1 by IHQ (2–3 immunoscore) although it did so in the ITT population. However, the study showed that the median duration of response (mDOR), a secondary endpoint, for those receiving atezolizumab was 21.7 months (95% CI 13.0, 21.7) in the overall study population, compared to 7.4 months (95% CI 6.1, 10.3) for those receiving chemotherapy. At the time of data cutoff, the majority (63%) of the subjects who responded to treatment with atezolizumab continued to respond, versus 21% of those treated with chemotherapy [61].

#### Recommendations

In cases that progress following platinum-based therapy, PD-1/PD-L1 inhibitors are standard options: pembrolizumab (level of evidence: 1. Grade of recommendation: A) and nivolumab or atezolizumab (Level of evidence: 2. Grade of recommendation: A).

Treatment with vinflunine is an alternative for patients in whom anti PD-1/PD-L1 therapy is not possible: Level of evidence: 1. Grade of recommendation: B.

Early palliative care is strongly recommended.

## Treatment after failure to respond to immune checkpoint inhibitors

Several immune checkpoint inhibitors have been approved for metastatic urothelial carcinoma over the last two years, both in platinum-refractory patients and in first line for cisplatin-unfit population, on the basis of phase II and phase III clinical trial results [51, 52, 58–61]. However, despite some exceptional responders who appear to derive long-term benefit, most will progress.

No randomized data exist regarding patients progressing to immune-oncology (IO) agents. The only data published are from a small, retrospective dataset with 62 patients [62] and reveal that a mere one-third of the cases with advanced UC are able to receive systemic therapy after PD-1/PD-L1 inhibitors. However, it must be pointed out that these subjects achieve similar outcomes to those historically observed in patients who had not received prior treatment with immune checkpoint inhibitors. Moreover, some patients show excellent radiological responses to chemotherapy [63], suggesting that at least a subset of cases will benefit from further treatment with chemotherapy after failing on IO agents.

### Recommendations

Close follow-up is imperative to initiate chemotherapy rapidly in those patients. If IO has been used in first-line, systemic therapy should follow the same rules as for treatment naïve patients, with cisplatin-based chemotherapy as the cornerstone of therapy. If administered in second line, vinflunine-based chemotherapy seems adequate if available; otherwise taxane-based chemotherapy: Level of evidence: II. Level of recommendation: A.

## New drugs in research

The signaling component of the Fibroblast Growth Factor (FGF) family comprised of eighteen secreted proteins that interact with four signaling tyrosine kinase FGF receptors (FGFRs1-4). Activated FGFR phosphorylate-specific tyrosine residues that mediate interaction with cytosolic adaptor proteins and the RAS-MAPK, PI3K-AKT, PLC, and STAT intracellular signaling pathways. Aberrant activity of the pathway is associated with developmental defects that disrupt organogenesis, impair the response to injury, and result in metabolic disorders, and cancer [64].

In the open-label, phase II study BLC2001 presented at the 2018 ASCO Annual Symposium [65], erdafitinib, an oral pan-FGFR tyrosine kinase inhibitor, was tested in 96 patients with metastatic or unresectable urothelial carcinoma and FGFR alterations (mutation in FGFR3 or fusion in FGFR2 or FGFR3). There was a 42% confirmed ORR (3% CR, 39% PR) and 80% disease control rate (CR + PR + SD). Similarly, among patients with prior immune checkpoint inhibitors (ICI) (*n* = 21), ORR was 70%. Preliminary data from the trial indicate a median overall survival of 13.8 months. Based on these outcomes, erdafitinib received breakthrough therapy designation by the FDA in June 2018. A Phase III is ongoing (THOR study: NCT02365597).

Others FGFR inhibitors are being tested in phase I/II clinical trials for advanced or metastatic UC alone or in combination with chemotherapy or ICI (Rogaratinib, B-701, AZD4547, BGJ398, Debio 1347, INCB054828, and LY3076226)

Tumor angiogenesis as an antineoplastic target for resistant UC is an old, but good treatment approach. In a phase III clinical trial, ramucirumab, a human IgG1 VEGFR-2 antagonist, plus docetaxel was compared to docetaxel plus placebo in 530 patients with advanced or metastatic UC who progressed during or after platinum-based chemotherapy [66]. The primary endpoint, PFS, was prolonged significantly in patients assigned to ramucirumab plus docetaxel versus placebo plus docetaxel [median 4.07 months (95% CI 2.96–4.47]) vs 2.76 months (2.60–2.96); HR 0.757; 95% CI 0.607–0.943; *p* = 0.0118]. A blinded independent central analysis was consistent with these results. Objective response was achieved by 53 (24.5%; 95% CI 18.8–30.3) of 216 patients assigned to receive ramucirumab and 31 (14.0%, 95% CI 9.4–18.6) of 221 assigned to placebo.

Antibody–drug conjugate (ADC) is a new therapeutic approach that combines the specificity of monoclonal antibodies, that recognized specific antigens on the tumor cell surface, and the cell-killing power of potent cytotoxic agents to treat cancer. In a phase I (EV-101) study, 155 patients with metastatic UC treated with ≥ 1 prior chemotherapy or who were ineligible for cisplatin received enfortumab vedotin (EV), an ADC that delivers a microtubule-disrupting agent (monomethyl auristatin E: MMAE) to tumors expressing Nectin-4, a protein overexpressed in most UCs. Updated results presented at the 2018 ASCO Congress [67], confirmed CR and PRs in 3 and 34 patients, respectively; ORR = 33% (95% CI 24.7–42.9). Overall median duration of response was 24.3 weeks (95% CI 16.3–47.3) and PFS was 23.1 weeks (95% CI 20.1–24.1). Median OS was 12.5 month (95% CI 8.1–14.8) with 76 patients (68%) censored and an OS rate at 6 months of 75.1%. Based on these results, enfortumab vedotin received breakthrough therapy designation by the FDA in June 2018. A Phase III is ongoing (EV-301 study; EudraCT: 2017-003344-21).

Another several ADCs against different UC surface markers are in the pipeline, but the ones that are furthest along include sacituzumab govitecan (IMMU-132: an anti-Trop-2 mAb conjugated with SN-38, the active metabolite of irinotecan), ASG-15ME (composed of a SLITRK6-specific human gamma 2 antibody conjugated to MMAE, a microtubule-disrupting agent) for advanced UC, and oportuzumab monatox (an anti-EpCAM humanized single-chain variable fragment linked to a truncated form of Pseudomonas exotoxin A) for early UC.
